# Enrichment of trimethyl histone 3 lysine 4 in the *Dlk1* and *Grb10* genes affects pregnancy outcomes due to dietary manipulation of excess folic acid and low vitamin B12

**DOI:** 10.1186/s40659-024-00557-3

**Published:** 2024-11-14

**Authors:** Divika Sapehia, Aatish Mahajan, Parampal Singh, Jyotdeep Kaur

**Affiliations:** Department of Biochemistry, Postgraduate Institute of Medical Education and Research, Chandigarh, India

**Keywords:** Vitamin B12, Folic acid, Placenta, Transgenerational, Histone methylation

## Abstract

The aberrant expression of placental imprinted genes due to epigenetic alterations during pregnancy can impact fetal development. We investigated the impact of dietary modification of low vitamin B12 with varying doses of folic acid on the epigenetic control of imprinted genes and fetal development using a transgenerational model of C57BL/6J mice. The animals were kept on four distinct dietary combinations based on low vitamin B12 levels and modulated folic acid, mated in the F0 generation within each group. In the F1 generation, each group of mice is split into two subgroups; the sustained group was kept on the same diet, while the transient group was fed a regular control diet. After mating, maternal placenta (F1) and fetal tissues (F2) were isolated on day 20 of gestation. We observed a generation-wise opposite promoter CpG methylation and gene expression trend of the two developmental genes *Dlk1* and *Grb10*, with enhanced gene expression in both the sustained and transient experimental groups in F1 placentae. When fetal development characteristics and gene expression were correlated, there was a substantial negative association between placental weight and *Dlk1* expression (r = − 0.49, p < 0.05) and between crown-rump length and *Grb10* expression (r = − 0.501, p < 0.05) in fetuses of the F2 generation. Consistent with these results, we also found that H3K4me3 at the promoter level of these genes is negatively associated with all fetal growth parameters. Overall, our findings suggest that balancing vitamin B12 and folic acid levels is important for maintaining the transcriptional status of imprinted genes and fetal development.

**Supplementary Information:**

The online version contains supplementary material available at 10.1186/s40659-024-00557-3.

## Introduction

From conception to the early stages of life to the age of two, these initial days of life are a critical time for nutrition. A child's prenatal development, from head to toe, can be severely hampered by a lack of vital nutrients during these phases. [1]. Vitamins are among the vital nutrients required for healthy and proper growth. The discovery that neural tube abnormalities are caused by a shortage of folate during the initial stages of pregnancy is a well-known example of how vitamins can affect fetal development [2]. Since folate deficiency is widely known to have adverse effects, many countries have started folic acid fortification in food [3]. However, cobalamin, often known as vitamin B12, which is also essential for intrauterine fetal development, has not gained that much significance [4]. If individuals with levels between 200 and 300 pg/mL are regarded as borderline deficient, then 47% of the population in India is vitamin B12 deficient. [5]. The recommended intake of vitamin B12 for women is 2.4 µg per day, which is increased to 2.6 µg per day during pregnancy to compensate for the daily requirement of a growing fetus. During pregnancy, the developing fetus absorbs nutrients, minerals, and vitamins through the maternal placenta. In vitamin B12-deficient pregnant mothers, the amount of vitamin transported to the fetus also decreases, which is the root cause of vitamin B12 deficiency in infants. In developed countries, nutritional vitamin B12 deficiency is relatively uncommon because of the high consumption and availability of animal-based foods [6]. However, in developing countries such as India, a vegetarian diet is preferred for religious and socioeconomic reasons, leading to vitamin B12 deficiency in a massive population. Children who are exclusively breastfed by deficient mothers are at risk of developing vitamin B12 deficiency, leading to low body weight and undernutrition at a very young age. In contrast to that of folic acid, the role of vitamin B12 deficiency in development and reproduction has not been well studied, even when it is known that these two vitamins are closely linked. Reports of vitamin B12 deficiency during pregnancy from worldwide have led to the recognition of vitamin B12 deficiency among women and children as a major health problem at the global level, which has rarely been addressed before [7–11]. The reported associations of vitamin B12 and folic acid with gestational outcomes have established the role of an imbalance of these vitamins in foetal abnormal growth as well as the development of insulin resistance in offspring [12, 13]; however, the underlying mechanism is not very well understood. Deficits or excesses in folic acid and vitamin B12 during early development can impair imprinting and induce developmental abnormalities because these nutrients have a role in the regulation of methylation through the one-carbon metabolism cycle. Any alterations in the imprinting and expression of even a single imprinted gene can cause aberrant growth and development.

*Dlk1* is one such imprinted gene expressed from the paternal allele. This gene encodes a transmembrane protein that regulates cell growth by including numerous repeats of the epidermal growth factor. Adipocytes are among the cell types whose differentiation is aided by this encoded protein. Previous studies have shown that *Dlk1* knockout mice are highly susceptible to perinatal lethality, pre-and postnatal fetal growth restriction followed by metabolic disorders, and obesity after birth [14], indicating that *Dlk1* plays a vital role in fetal growth promotion. Another gene in our study, *Grb10,* has unique imprinted tissue-specific expression in adult mouse tissues from the maternal allele and from the paternal allele  in a subset of neurons [15]. A previous study has also revealed overgrowth in multiple organs and tissues in *Grb10* knockout mice [16]. Therefore, in this study, we investigated the influence of an imbalance in parental dietary vitamin B12 and folic acid intake on the epigenetic regulation of *Dlk1* and *Grb10* in the placenta and their association with fetal development. We assessed the mRNA levels of these genes in placental and fetal tissues in a mouse model established for transgenerational studies. We also investigated the function of epigenetic processes in controlling the expression of the imprinted genes *Dlk1* and *Grb10*. We further validated regulation of expression of genes by the epigenetic mechanisms in a human placental cell line.

## Materials and methods

### Animal model and diets

For the F0 generation, a total of 64 C57BL6/J mice were utilized, with 12 females and 4 males in each group. After a week of feeding the animals a regular chow diet, the animals were split into four groups based on the dietary combinations of vitamin B12 and folic acid. Animals were fed with these semisynthetic dietary combinations (based on AIN93G with modulation of folic acid and vitamin B12) for four weeks—BNFN, where the required concentration of vitamin B12 (0.025 mg/kg) and folic acid (2 mg/kg) is present; BDFD, where both vitamins are deficient; BDFN, where vitamin B12 is deficient, and a normal folic acid concentration are present; and BDFO, where vitamin B12 is deficient but folic acid is oversupplemented (8 mg/kg). The composition of the diet is provided in Supplementary table 1. The animals were mated within the group after four weeks, and on the twentieth day of pregnancy, the pregnant females were sacrificed to harvest the placenta and fetal organs. At this stage, fetal growth characteristics, including head circumference, placental weight, fetal weight, and crown–rump length, were examined in isolated fetuses.

**Fig. 1:**
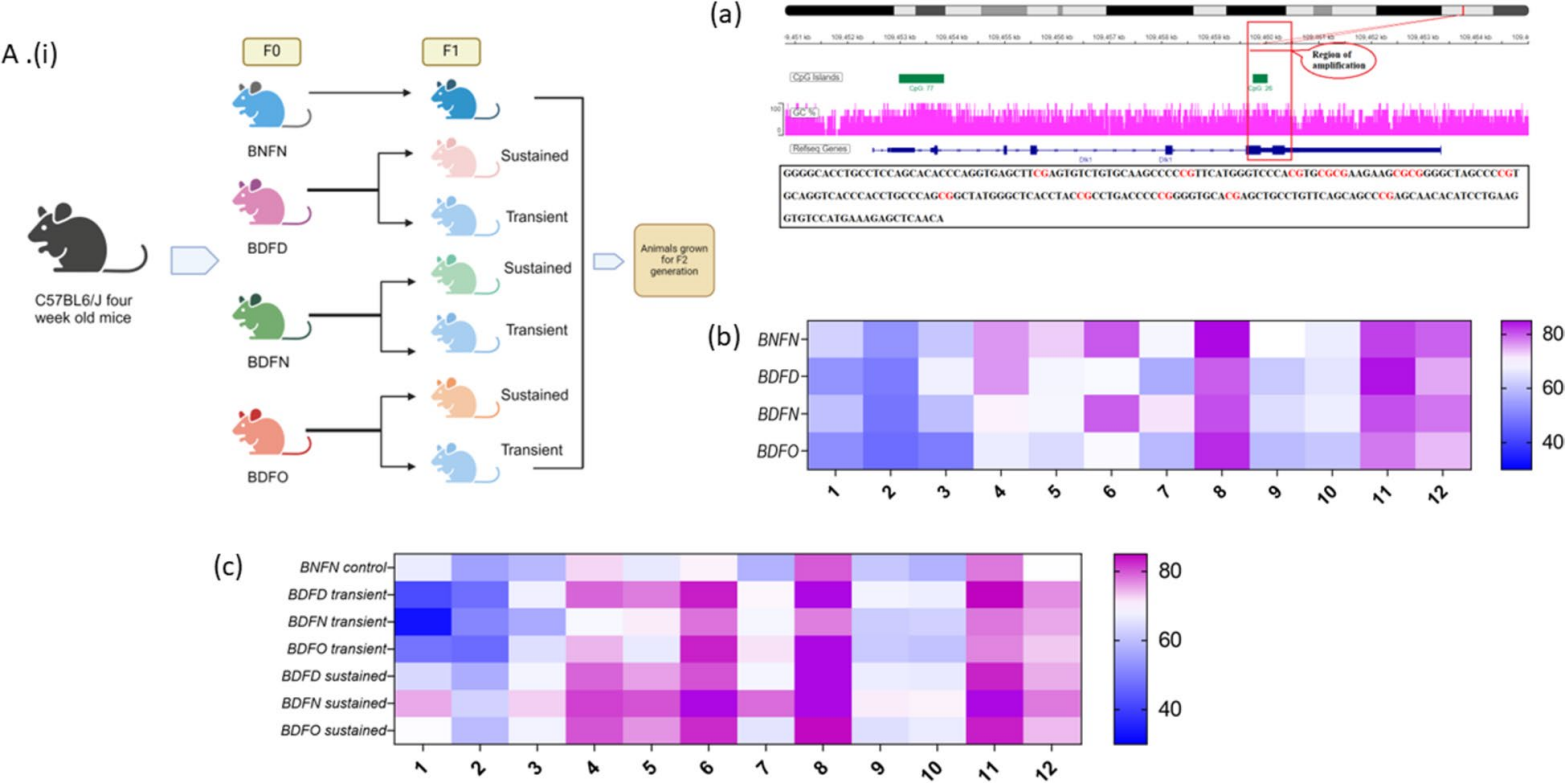
**A** (i) Transgenerational animal model used in the study. **a** Representative Integrative Genomic Viewer (IGV) tracks of a genomic sequence selected to study CpG methylation status in the *Dlk1* promoter region. Heatmap analysis showing the methylation pattern (percent methylation) of *Dlk1* in **b**. F0 and **c**. F1 generation

For the F1 generation, animals in the normal control group were fed the same diet, while those in the other test groups were divided into two sub dietary groups. In each subgroup, the animals continued on the same diet as their parents, denoted “sustained groups”, or they were shifted to a normal control diet, designated “transient groups.” In each group, the animals were mated after feeding for 6–8 weeks, and the pregnant females were subsequently sacrificed for tissue collection and observation of fetal growth parameters. Similar dietary groups were carried forward to the F2 generation, and blood was collected to estimate vitamin levels (Fig. 1A(i)).

### DNA Methylation analysis

Total DNA was isolated from the placental tissues of the F0 and F1 generations using a commercially available Qiagen QIAamp DNA isolation kit, and the quality of the DNA was quantified spectrophotometrically. The methylation of the promoter regions of genes was quantitatively analysed by bisulfite sequencing at CpG sites. The isolated DNA was then subjected to bisulfite conversion with a Qiagen kit. A quality check of bisulfite-converted DNA was performed again after conversion. The bisulfite-converted DNA around the promoter region of these genes was amplified with primers (Supplementary table 2). Amplified PCR products were sequenced using sequencing services provided by Eurofinn Pvt. Ltd. Chromatograms of the polymerase chain reaction products were analysed for C and T peak heights. Finally, the percentage of methylation at each CpG site was analysed.

### Histone  Methylation analysis

To investigate histone enrichment at the promoter region of these genes, a chromatin immunoprecipitation assay was used. Three types of histone methylation were studied, two repressing H3K9me3 and H3K27me3 and one activating H3K4me3. The ChIP assay was performed according to the previous protocol of Rahat et al. [17]. Antibodies against trimethyl H3-K4/K9/K27 and normal rabbit immunoglobulin G (IgG) (Abcam) were used for immunoprecipitation. The data were normalized to the input DNA, and the fold enrichment was calculated relative to nonspecific antibodies. The sequences of primers used are listed in Supplementary table 3.

### Gene expression analysis

Placenta and fetal tissues (brain and liver) collected after sacrificing pregnant females were used to estimate mRNA levels. Total RNA was isolated by the TRIzol method, and the extracted RNA was converted to cDNA using a commercially available kit (Bio-Rad, USA). Primers were designed for two imprinted genes, *Dlk1* and *Grb10* (Supplementary table 4), and mRNA levels were quantified using real-time PCR. Primers were designed for mRNA expression study using Primer-BLAST online available NCBI tool (http://www.ncbi.nlm.nih.gov/tools/primer-blast/). Primers designed by Primer-BLAST were cross- checked for primer self-dimerization and potential hairpin formation in online available software, Oligonucleotide Properties Calculator (http://www.basic.northwestern.edu/BIOTOOLS/OLIGOCALC.HTML) and were made to BLAST in NCBI database. (http://www.basic.northwestern.edu/BIOTOOLS/OLIGOCALC.-HTML). After quantitative real- time PCR, the PCR products were run on agarose gel for further confirmation. The results were calculated using β-actin as an internal control.

### Cell culture studies with vitamin modulations

The human placental cell line HTR8/SVneo was obtained from ATCC (USA). Cell lines were cultured in Dulbecco’s modified Eagle’s medium (DMEM-D2429), which is deficient in folic acid and vitamin B12. The growth medium was supplemented with 10% FBS and 1% antibiotic–antimycotic solution. The cells were maintained at 37 °C in 95% humidity and 5% CO_2_. The growth rates were determined by cell counting. The cells were serum starved overnight in serum-free media before any of the experimental procedures described below. According to the content of folic acid and vitamin B12 in the culture medium, the cells were divided into four treatment groups, as shown in Table 1. Cells in the control and test groups were subjected to vitamin B12 and folic acid treatments for 48 h.

**Table 1:** Vitamin B12 and folic acid levels in cell culture studies

Sr no.	Name	vitamin combinations	Vitamin B12 concentration	Folic acid concentration
Group 1	BNFN	Vitamin B12 normal and folic acid normal	100 ng/mL	20 ng/mL
Group 2	BDFD	Vitamin B12 deficient folic acid deficient	0	0
Group 3	BDFN	Vitamin B12 deficient folic acid normal	0	20 ng/mL
Group 4	BDFO	Vitamin B12 deficient and folic acid over supplemented	0	2000 ng/mL

### 5-Azacitidine treatment and gene expression

To determine whether the expression of genes under study is epigenetically regulated by an altered ratio of vitamin B12 and folic acid, HTR8/SVneo cells were treated with 5-azacitidine at a concentration of 5 μM for 24 h [18]. We measured the expression levels of genes before and after 5-azacitidine treatment and analysed whether treatment with 5-azacitidine alters the expression of genes. 5-Azacitidine acts by inhibiting the enzyme DNA methyltransferase 1 (DNMT1), which methylates cytosine residues in eukaryotic DNA. RNA was isolated from the treated and untreated cells. The expression of the *Dlk1 and Grb10* genes was analysed by quantitative real time‒PCR (the primers used are listed in Supplementary Table 5).

### Statistical analysis

ANOVA (one-way) and post hoc Tukey tests were used for statistical analysis to compare the dietary groups. Graph plotting and analysis were performed using GraphPad Prism (v.8.0.1). Correlations between several parameters were determined using Pearson’s correlation analysis. The data are displayed as the mean ± standard deviation, with p < 0.05 indicating statistical significance.

## Results

A C57BL6/J mouse model was used to study transgenerational epigenetics for three generations: F0, F1, and F2. Along with the control diets, animals in the F0 generation were fed different dietary combinations containing vitamin B12 and folic acid. To investigate the in utero effect of the dietary combinations, the animals were sacrificed on the twentieth day of gestation to collect placental and fetal tissues and to evaluate other parameters, such as placental weight, fetal weight, head circumference, and crown–rump length. Similar parameters were repeated for the next generation for the transient and sustained dietary groups, as described in the *Materials and Methods section (Animals and diets*). Pups exposed to vitamin B12 deficiency and an excess folic acid diet through their mothers (F0) showed a significant reduction in the abovementioned parameters. Suppression of these parameters was also observed in the next generation, exclusively in the BDFO transient groups, where the animals were exposed to an imbalanced diet in the previous generation and were subsequently shifted to the control diet [19].

### Distinct patterns of CpG methylation in the promoter regions of imprinted genes

**Fig. 2:**
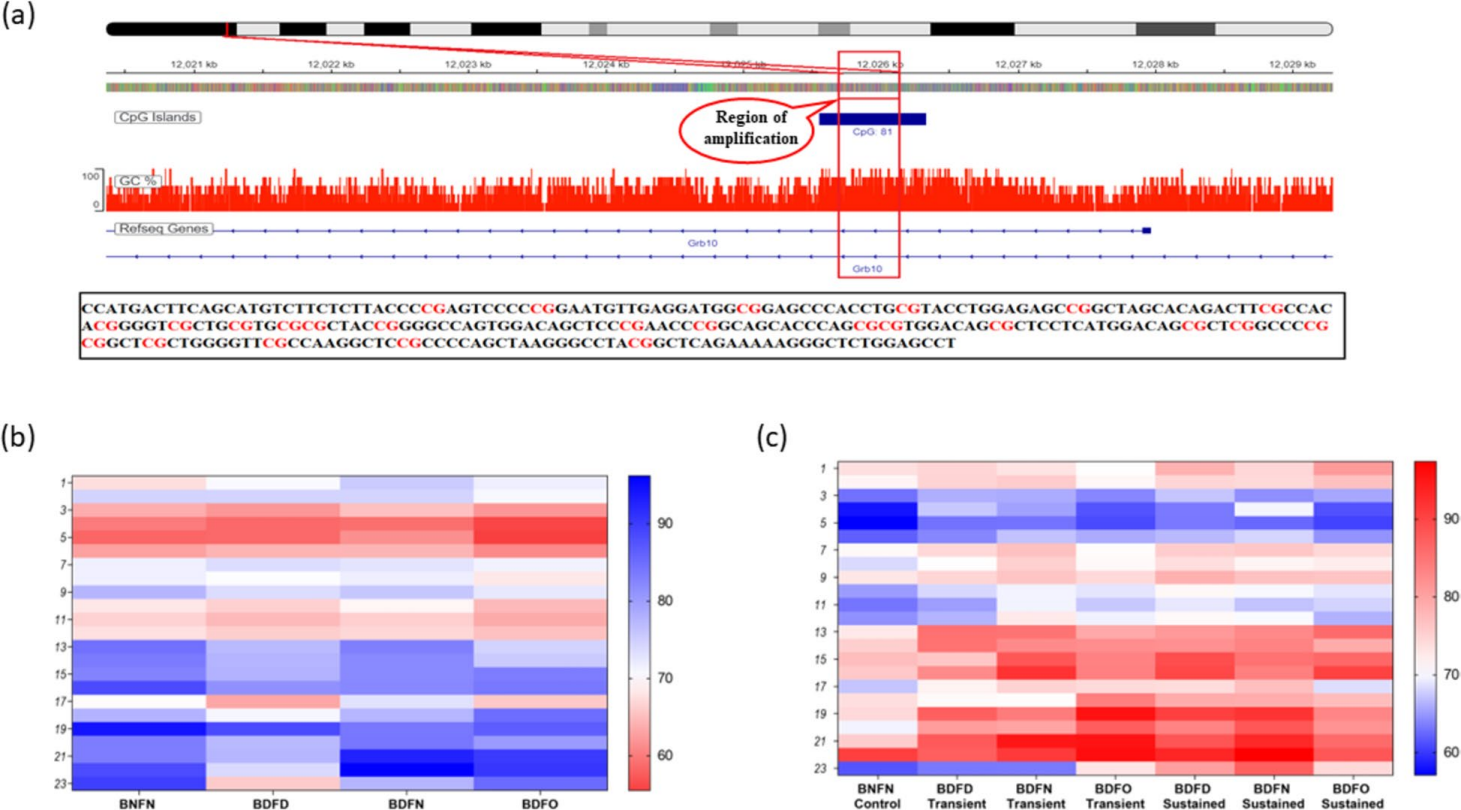
**a** Representative Integrative Genomic Viewer (IGV) tracks of a genomic sequence selected to study CpG methylation status in the *Grb10* promoter region. Heatmap analysis showing the methylation pattern of *Grb10* in (**b**). F0 and **c** F1 generation

To investigate the role of DNA methylation as a regulatory mechanism for *Dlk1-Grb10* imprinted genes in fetal development, the methylation of 12 CpG sites in the region present around the 201 base pair long promoter of the *Dlk1* gene (Fig. 1a) and 23 CpG sites in the region present around the 279 base pair long promoter of *Grb10* was analysed in placental tissue (Fig. 2a).

In the F0 generation, a hypomethylated CpG island was detected in the promoter region of *Dlk1* after mice were fed different combinations of diets compared to the control group. As shown in Fig. 1b, all the dietary groups in the F0 generation showed decreased methylation at almost all the CpG sites studied. Under transient conditions in the F1 generation, however, an increase in methylation was found at CpG sites 4 to 8 and 11 in the promoter region of *Dlk1* in the BDFD and BDFO groups compared to the control group (Fig. 1c). In sustained dietary combinations, the trend in methylation change was quite different from that in the F0 generation. Compared with those in the control group, the animals in these groups exhibited hypermethylation at the maximum CpG sites (Fig. 1c).

For the *Grb10* gene, in generation F0, we found a decrease in the methylation of the *Grb10* gene from CpG sites 8 to 23 in the BDFD group compared to the BNFN group. A decrease in methylation was also detected at a stretch of CpG sites 2–17 in the BDFO group, whereas the BDFN group was hypomethylated at CpG sites 16, 19, and 23. Overall, a hypomethylated promoter region similar to that in *Dlk1* was found in all the dietary groups in the F0 generation (Fig. 2b). In the F1 generation, more methylation from CpG sites 3 to 23 was detected in all the transient dietary groups than in the control group, except at a few CpG sites. Under sustained circumstances, a similar trend of hypermethylation to that under transient conditions was observed in all three groups. Hypermethylation was detected at all CpG sites in the BDFO sustained group, with a high percentage of methylation observed at CpG numbers 13 (13%), 15 (9%), 19 (9%), 20 (12%), 21 (10.4%) and 23 (12.8%). At the same CpG sites, methylation levels increased to 19 (16%), 20 (14%), and 21 (13%) in the BDFD-susceptible group and 19 (17%) and 20 (18%) in the BDFD-susceptible group (Fig. 2c).

### Transient dietary conditions lead to the enrichment of H3K4 methylation marks on promoter regions of imprinted genes in vitamin B12-deficient and folic acid oversupplemented groups

**Fig. 3:**
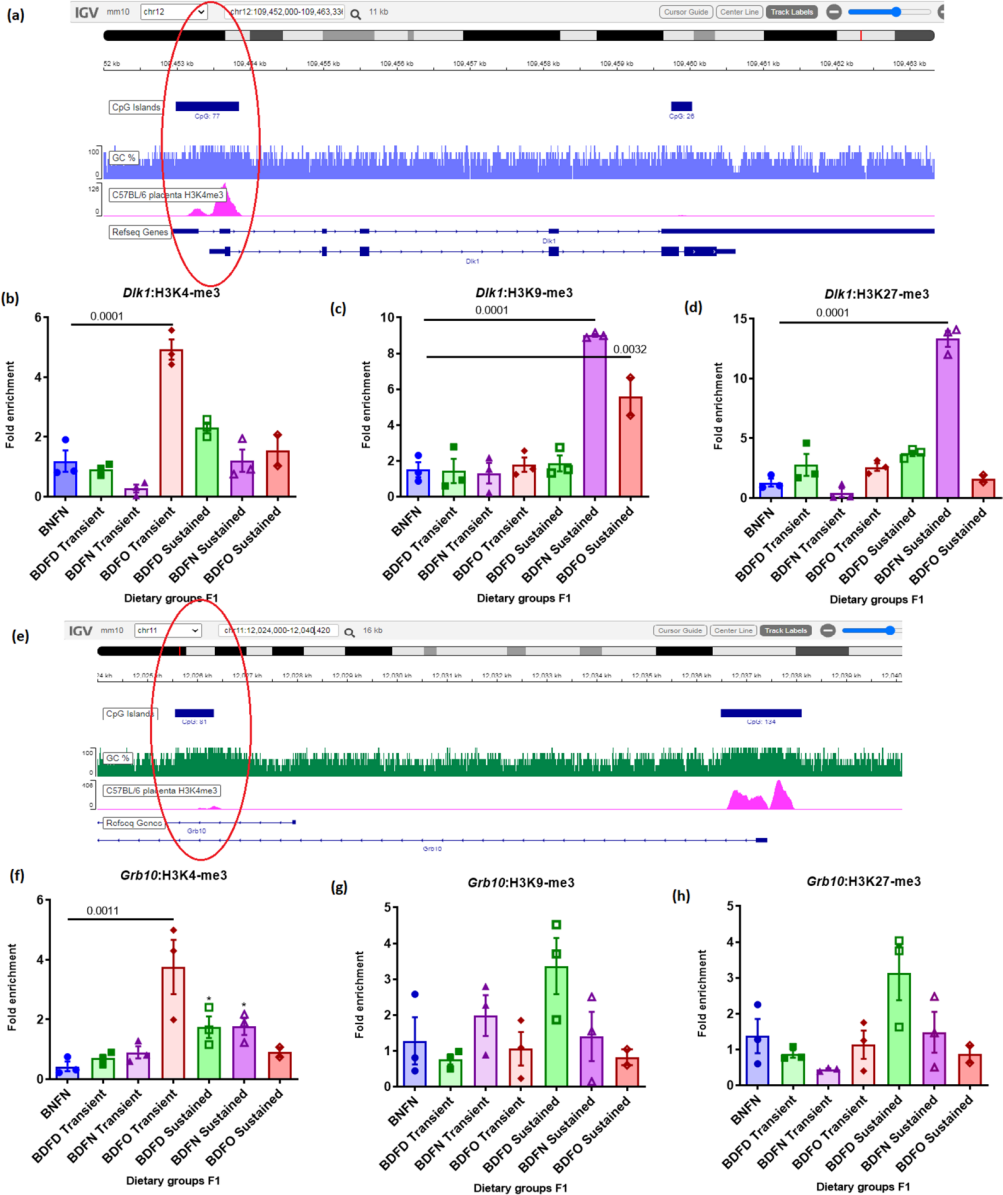
Representative Integrative Genomic Viewer (IGV) tracks of the genomic region were selected to study histone methylation in the **a** *Dlk1* and **e** *Grb10*promoters. Quantification of **b** and **f** H3K4me3, **c** and **g** H3K9me3, and **d** and **h** H3K27me3 at the promoter regions of *Dlk1 and Grb10* in placental tissues of the F1 generation. The fold enrichment relative to nonspecific IgG was used as a negative control and was normalized to that of the input DNA. ∗  ∗ *p* < 0.01 and ∗  ∗  ∗ *p* < 0.001 vs. BNFN. The data are presented as the mean of the observed fold change ± SD. The values are presented as the means ± SDs of n = 3 except for *n* = *2* for the sustained BDFO group in the F1 generation

By examining histone methylation at the vicinity of the promoter of imprinted genes through ChIP‒qPCR (in placental tissue of the F1 generation; Fig. 3a and e), H3K4-me3 levels were found to significantly increase at the *Dlk1* promoter in the BDFO transient group (4.9-fold; p =0.0001), and H3K4-me3 levels decreased with other transient conditions, such as BDFD and BDFN. In the sustained diet group, there was an increase in H3K4 trimethylation in all the groups—BDFD (2.3-fold), BDFN (1.2-fold), and BDFO (1.5-fold) sustained groups—but not significantly (Fig. 3b). The enrichment of H3K9 trimethylation, a repressive histone marker, was significantly greater in the BDFN- (9fold; p = 0.0001) and BDFO- (5.5-fold; p =0.0032) sustained groups than in the control group, while no significant increase was found in the dietary groups under transient conditions (Fig. 3c). Increased levels of H3K27me3, another repressive modification, were detected in the BDFN-susceptible group of *Dlk1* (13-fold; p < 0.001), while no significant changes were detected in the other dietary conditions (Fig. 3d).

Estimation of the tri-methylation of H3K4, the activation mark in the promoter region of *Grb10,* revealed that the levels were significantly increased in the BDFO transient group (4.6-fold; p = 0.0011) (Fig. 3f). Under sustained conditions, there was an increase in histone trimethylation in the sustained BDFO, BDFD (1.74-fold; p < 0.05) and BDFN (1.7-fold; p < 0.05) groups compared to those in the control group. The levels of the repressive marks H3K27 and H3K9 trimethylation in the *Grb10* promoter region were in the BDFD group under sustained conditions than in the control group (Fig. 3g and h).

### Expression of imprinted genes in two generations

**Fig. 4:**
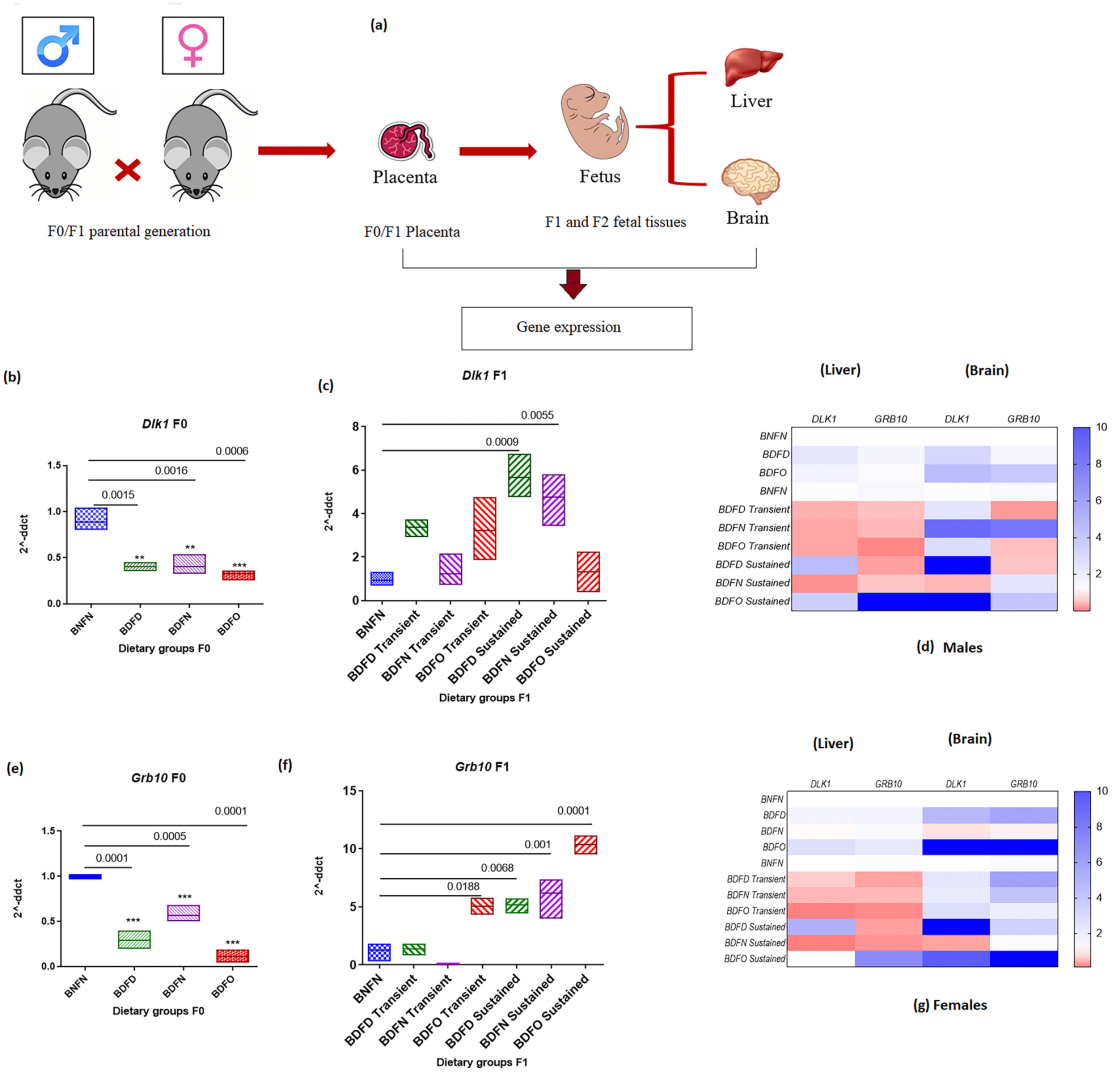
**a** Schematic of the experimental design used for the study. mRNA expression of *Dlk1* in the **b** F0 and **e** F1 generations and the expression of *Grb10* in the **c** F0 and **f** F1 generations in mouse placental tissue normalized to the housekeeping gene β-actin. The values are presented as the means ± SDs of n = 3 except for *n* = *2* for the sustained BDFO group in the F1 generation. The X-axis represents different dietary groups. ∗ *p* < 0.05, ∗  ∗ *p* < 0.01, ∗  ∗  ∗ *p* < 0.001 vs. control. Heatmap analysis of the mRNA levels of *Dlk1* and *Grb10* in the liver and brain fetal tissues of **d** males and **g** females

After assessing the changes in promoter CpG methylation and histone methylation in the placental *Dlk1* and *Grb10* genes in the placental tissues of the two generations of mice, we next investigated the gene expression levels in placental and fetal tissues (Fig. 4a)*.*

(i) Expression analysis of *Dlk1* and *Grb10* in the placenta: In F0 placenta, the mRNA levels of *Dlk1* in the placenta were downregulated in all the groups compared with those in the control group (Fig. 4b). BDFO (0.32-fold; p = 0.006), BDFD (0.4-fold; p = 0.0015) and BDFN (0.4-fold; p < 0.0016). In contrast to those in the previous generation, in the F1 generation, the mRNA levels of *Dlk1* tended to increase in all the transient groups, albeit not significantly, and significantly increased in the BDFD (5.6-fold; p =0.0009) and BDFN (4.7-fold; p = 0.0055) sustained groups in comparison to those in the control group (Fig. 4c). The expression of the *Grb10* gene in the maternal placenta also decreased in each group of the F0 generation (Fig. 4e), viz. BDFD (0.29-fold; p = 0.0001), BDFN (0.58-fold; p = 0.0005) and BDFO (0.11-fold; p = 0.0001). *Grb10* expression in the F1 generation, similar to that in the *Dlk1* generation, showed the opposite trend; that is, compared to that in the control group, there was significantly greater expression in the BDFO transient group (5.05-fold; p = 0.0188) and all sustained dietary groups, including the BDFD (5.1-fold; p = 0.0068), BDFN (6.1-fold; p = 0.001), and BDFO (10.3-fold; p = 0.0001) groups (Fig. 4f).
(ii) Expression analysis of *Dlk1* and *Grb10* in fetal tissues: To observe the sex- and tissue-specific effects of dietary modulation, the mRNA levels of *Dlk1* and *Grb10* were also analysed in fetal tissues (liver and brain Fig. 4d and g).

- *Dlk1* expression in the fetal liver: In the liver, in the F1 generation, in females, the BDFO group exhibited highly increased *Dlk1* expression (2.68-fold; p < 0.001), whereas in males, significantly (2.28-fold, p < 0.05) increased Dlk1 expression was detected in the BDFD group. In the F2 generation, both males and females experienced a significant decrease in the relative expression of the *Dlk1* gene in the transient groups. Under sustained conditions, however, a significant increase was found in the BDFO (male, 3.58-fold; p < 0.001) and BDFD (male, 4.52-fold; female, 5.2-fold; p < 0.001) groups.
- Expression of *Dlk1* in the fetal brain: In the fetal brain, in comparison to those in the control group, the mRNA levels of *Dlk1* imprinted genes were found to increase under both vitamin deficiency conditions (BDFD) in the F1 generation in both males and females; in the fetal brain tissue of the F2 generation, the expression of Dlk1 in the fetal liver tended to significantly increase (p < 0.001), similar to that in the fetal liver in the BDFO sustained (male 13-fold; p < 0.001, female 9.76-fold; p < 0.001) and BDFD sustained (male 16.8-fold; p < 0.001, female 12.02-fold; p < 0.001) groups; and in both males and females, Dlk1 expression decreased (male 0.4-fold & female 0.36-fold) in the BDFN sustained group.
- Expression of *Grb10* in the fetal liver: Estimating the levels of *Grb10* mRNA in the fetal liver in the F1 generation, the levels were found to be significantly increased only in female fetuses under both vitamin deficiency conditions, i.e., BDFD (1.69-fold; p < 0.01), and in vitamin B12-deficient folate-supplemented conditions, i.e., BDFO (2.2-fold; p < 0.001). In the F2 generation, *Grb10* gene expression was significantly upregulated in plants supplemented with vitamin B12-deficient folate, i.e., BDFO (male, 14.9-fold; female, 71.9-fold; p < 0.001) sustained conditions in both males and females.
- Expression of *Grb10* in the fetal brain*:* Compared with that in the control group (BNFN), the expression of *Grb10* in the fetal brain of the F1 generation was significantly greater in the BDFD (male, 1.45-fold and female, 5.70-fold; p < 0.001) and BDFO (male, 3.92-fold and female, 13.73-fold; p < 0.001) groups in both males and females. In the F2 generation, in males, there was a significant increase in *Grb10* expression in the BDFN transient group (8.29-fold; p < 0.01) and the BDFO sustained group (4.0-fold: p < 0.05), whereas in females, the expression increased significantly in the BDFD transient group (5.9-fold; p < 0.01) and the BDFO sustained group (26.9-fold; p < 0.001).

### Activating H3K4me3 is important for the transcriptional activation of imprinted genes

**Fig. 5:**
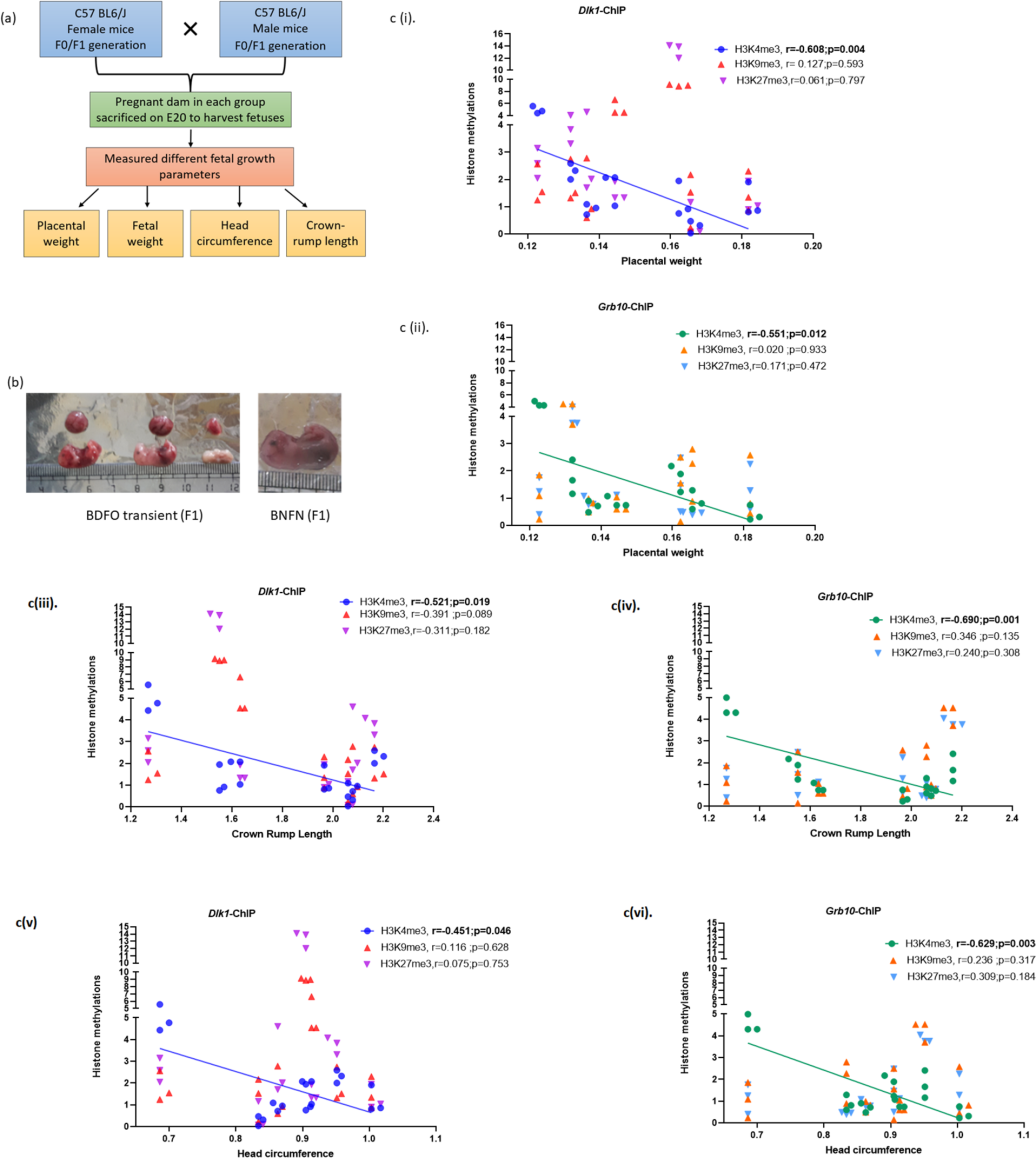
**a** Schematic of the experimental design and mating strategy used for the study. **b** Representative image of fetuses born in the F1 generation in the BNFN and BDFO transient dietary groups. **c** Graphs showing the correlation between histone methylation and foetal growth parameters. c (i-ii) Correlations of histone enrichment at the (i) *Dlk1* and (ii) *Grb10* promoters with placental weight. Correlation of histone enrichment at the *Dlk1* and *Grb10* promoters with (iii, iv) head circumference and (v, iv) crown rump length. Promoter region selected for different histone modification is around the CpG islands in the vicinity of promoter region Dlk1—chromosome number 11-12,025,555 and for Grb10 Chromosome number 12-109452966-109453847

After studying histone methylation, we then tried to correlate growth parameters with gene expression and histone methylation (Table 2 and 3) . *Dlk1 and Grb10* were significantly positively correlated with placental weight (r = 0.79, p < 0.01-*Dlk1*; r = 0.824, p < 0.001-*Grb10*) and crown-rump length (r = 0.57, p < 0.05-*Dlk1*; r = 0.873, p < 0.001-*Grb10*) in F0 placenta. However, fetal weight and head circumference were not significantly correlated with the expression of these mRNAs. In contrast, *Dlk1* gene expression was negatively correlated with placental weight in the following generation (F1) (r = − 0.49, respectively, p < 0.05), and *Grb10* gene expression was also negatively associated with crown-rump length (r = − 0.501, p < 0.05) in fetuses of the F2 generation (Fig. 5c(iv)). The remaining growth parameters did not correlate significantly with any of the genes (Table 3).

**Table 2:** Pearson correlation analysis for expression in placenta (F0) and fetal growth parameters (F1)

mRNA expression	Fetal weight	Placental weight	Head circumference	Crown-rump length
*Dlk1*	0.386 (0.215)	0.796^**^ (0.002)	0.234 (0.463)	0.586^*^ (0.045)
*Grb10*	0.228 (0.476)	0.824^**^ (0.001)	0.482 (0.113)	0.873^**^ (0.001)

Value represents: -Pearson coefficient (p-value)

^*^Correlation is significant at the 0.05 level (2-tailed)

^**^Correlation is significant at the 0.01 level (2-tailed)

**Table 3:** Pearson correlation analysis for expression in placenta (F1) and fetal growth parameters (F2)

mRNA expression	Fetal weight	Placental weight	Head circumference	Crown-rump length
*Dlk1*	− 0.005 (0.984)	− 0.491^*^ **(0.028)**	− 0.136 (0.569)	− 0.030 (0.901)
*Grb10*	0.023 (0.922)	− 0.326 (0.161)	− 0.048 (0.842)	− 0.501^*^ **(0.025)**

Value represents: -Pearson coefficient (p-value)

^*^Correlation is significant at the 0.05 level (2-tailed)

The significant correlation values are highlighted with the bold text

Additionally, correlation coefficients were studied to determine the relationships between clinically relevant fetal growth measures and histone alterations (methylation) in the *Dlk1* and *Grb10* promoters Table 4 and Fig. 5. Significant negative correlations were detected between H3K4me3 at the *Dlk1* gene promoter and placental weight (r = − 0.61, p = 0.004), head circumference (r = − 0.45, p = 0.0046), and crown-rump length (r = − 0.52, p = 0.019) (Fig. 5c(i, iii, v)). Additionally, there was a significant negative correlation between the activating promoter H3K4me3 of the *Grb10* gene and, placental weight (r = − 0.55, p = 0.0012) (Fig. 5c(vi)), head circumference (r = − 0.63, p = 0.003) (Fig. 5c(iv)) and crown–rump length (r = − 0.69, p = 0.001) (Fig. 5 vi).

**Table 4:** Pearson correlation analysis for histone modifications and fetal growth parameters

	Fetal weight	Placental weight	Head circumference	Crown-rump length
*Dlk1*				
H3K4me3	− 0.37 (0.10)	− 0.61^**^ **(0.004)**	− 0.45^*^ **(0.05)**	− 0.52^*^ **(0.02)**
H3K9me3	0.12 (0.60)	0.13 (0.59)	0.12 (0.63)	− 0.39 (0.09)
H3K27me3	0.13 (0.59)	0.06 (0.80)	0.08 (0.75)	− 0.31 (0.18)
*Grb10*				
H3K4me3	− 0.48^*^ **(0.03)**	− 0.55^**^ **(0.01)**	− 0.63^**^ **(0.003)**	− 0.69^***^ **(0.001)**
H3K9me3	0.16 (0.51)	− 0.02 (0.93)	0.24 (0.32)	0.35 (0.14)
H3K27me3	0.28 (0.23)	− 0.17 (0.47)	0.31 (0.18)	0.24 (0.31)

Value represents: -Pearson coefficient (p-value)

^*^Correlation is significant at the 0.05 level (2-tailed)

^**^Correlation is significant at the 0.01 level (2-tailed)

^***^Correlation is significant at the 0.001 level (2-tailed)

The significant correlation values are highlighted with the bold text

### 5-aza restored gene expression and methylation patterns in cells exposed to excess folic acid and vitamin B12 deficiency.

**Fig. 6:**
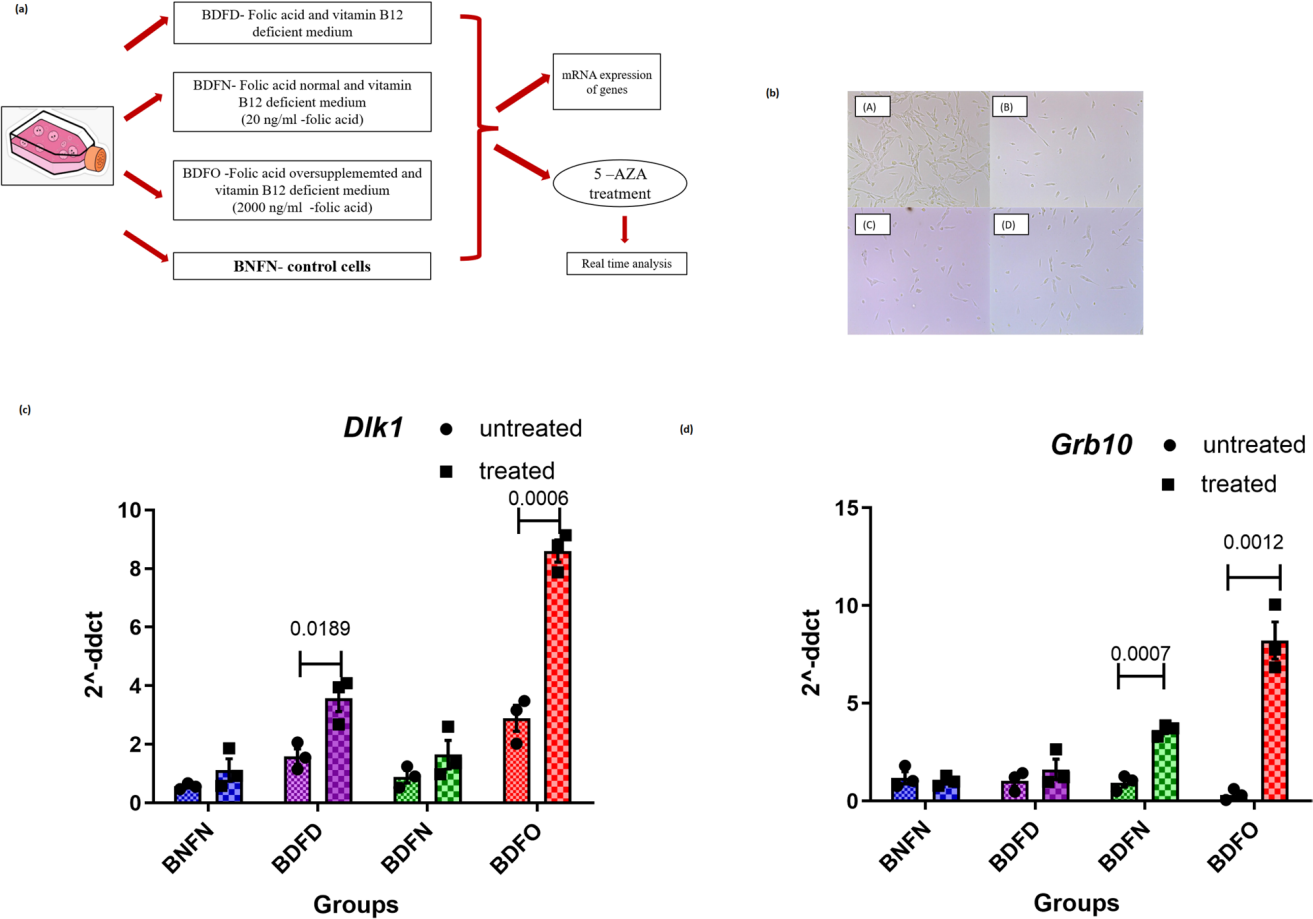
**a** Schematic of the experimental design for the cell culture experiment. **b**. Growth of placental cells in different groups after 48 h: BNFN (control), (A) BNFN, (B) BDFD and (C) BDFN (D) BDFO. The expression of **c** *Dlk1* and **d** *Grb10* in the placental cell line HTR8/SVneo with and without drug treatment was normalized to that of the internal control β-actin. The X-axis represents different vitamin combinations. *p < 0.05, **p < 0.01, ***p < 0.001 vs. control

HTR8/SVneo cells that were trophoblastic in origin and had an epithelial-like morphology were treated with the 5-azacitidine (Fig. 6a). Changes in the cellular morphology associated with a reduction in the number of HTR8/SVneo cells were observed upon treatment with 5-azacitidine in different groups (Fig. 6b). *Dlk1* expression was significantly upregulated by 3.5-fold (p = 0.0006) in the vitamin B12-deficient folate oversupplemented-treated group compared with BDFO untreated group (Fig. 6c). In addition, cells in both vitamin deficiency groups (BDFD) also showed a significant increase in the expression of the *Dlk1* gene by 2.3-fold (p = 0.0189) upon treatment with 5-azacitidine compared to that in untreated cells in the same group. However, drug treatment did not affect significantly *Dlk1* expression in BNFN or BDFN cells (Fig. 6c). For *Grb10* gene expression, a 4-fold (p = 0.0007) increase in the BDFN group and a 25.3-fold (p = 0.0012) increase in the BDFO group were observed after treatment of the cells with the drug compared to the untreated conditions in the respective groups. However, there was no difference in *Grb10* expression between the BNFN and BDFD groups, similar to what was observed for *Dlk1* (Fig. 6d). Hence, vitamin B12 deficiency in the presence of deficient and overfolic acid increased the levels of imprinted genes upon DNA demethylation, suggesting a role for epigenetic regulatory mechanisms (Table 4).

## Discussion

The influence of diet on epigenetics in genomic studies has provided many important insights into the roles of imprinted genes in establishing and maintaining epigenetic marks during pregnancy. The methylation of these genes is largely regulated by levels of folic acid and vitamin B12 in the diet. In addition to this, an imbalance of these vitamins can impact the imprinting of developmental genes. In the presence of B12 deficiency, 5-methyltetrahydrofolate (5MTHF) becomes physiologically imprisoned which hinders total folate metabolism. The reason for this trap is that 5MTHF cannot be converted back to methylenetetrahydrofolate, its precursor, or metabolized through the methionine synthase pathway. In the event of B12 deficiency and high folate due to a disturbed methionine cycle, the levels of S-adenosyl methionine get deranged leading to the epigenetic alterations in the genes [20].

We aimed to increase our understanding of the methylation of placental imprinted genes by first quantitatively examining the dynamic range of CpG sites and histone methylation across the promoters of genes expressed in the placenta, followed by the expression of genes whose expression changes in response to parental vitamin B12 deficiency with the modulation of folic acid levels. Our results suggest that dietary manipulation of low vitamin B12 and high folic acid during the period of reprogramming changes the methylation status and expression levels of imprinted genes in placental cells that are subjected to fetal development. H3K4 trimethylation appears to contribute to establishing an activating state of fetal growth-specific imprinted genes.

We detected placental hypomethylation at the studied CpG sites in *Dlk1* in different dietary groups of the F0 generation, with downregulated Dlk1 expression, which suggested that methylation of CpG islands in this promoter region has a limited regulatory effect on the expression of *Dlk1*. However, methylated CpG at promoter sites in the placenta can be a regulatory factor in determining *Dlk1* expression, as evidenced by previous studies by Zhao et al*.*, even though their findings were limited to GDM patients. Nevertheless, their research indicated that it is critical to understand the epigenetic modification of the imprinting gene *Dlk1*, as this modification may be a possible mechanism underlying fetal programming of growth [21]. According to a different study, the Dlk1/Meg3 region is hypomethylated in children with temple syndrome, a condition marked by pre- and postnatal growth retardation, feeding difficulties, and motor development delay [22]. In addition to *Dlk1,* the promoter methylation of *Grb10* in F0 maternal placenta decreased by 5–20% under the different dietary conditions compared to that of the control, except at a few CpG sites. Nakamura et al*.* reported that *Grb10* hypomethylation was associated with an overgrowth phenotype in a Japanese boy [23]. In another study by Bak et al*.,* an imprinting disorder marked as transient neonatal diabetes mellitus 1 (TNDM1), characterized by the presence of IUGR and diabetes mellitus, was caused by hypomethylation at imprinted loci at *Grb10* [24]. Nilsson et al*.* also reported that differential methylation of *Grb10* can be associated with type 2 diabetes [25].

In our study, in contrast to those of the F0 generation, the placenta of the F1 generation displayed a distinct pattern of expression in response to dietary modifications, where mostly sustained dietary groups exhibited enhanced expression. Additional investigation of promoter region methylation in the placental tissue of the F1 generation showed that there was more methylation in both the transient and sustained groups than in the control group. Since the increased expression and higher methylation levels of the placenta in the F1 generation did not correlate, alternative regulatory mechanisms are likely responsible for the increased transcriptional activity of the Dlk1 gene. In the case of *Grb10,* both the transient and sustained groups of the F1 generation also showed higher levels of methylation at several CpG sites. A study by Chang et al*.* reported that hypermethylation at *Grb10* is associated with congenital heart disease [26], whereas another study related to vitamin D levels reported that methylation changes were distinct between two subsequent generations around the imprinted domain of *Grb10* and *Dlk1* [27]*.*

To further investigate the contributing factors regulating gene expression in response to alterations in dietary folic acid and vitamin B12, we analysed the different histone methylations. We observed considerably increased occupancy of H3K4me3 in the BDFO transient group and of H3K9me3 and H3K27me3 in the BDFN sustained group in the *Dlk1* gene promoter. The increased expression of the *Dlk1* gene in the F1 placenta in the BDFO transient and BDFD sustained groups can be attributed to the enrichment of the activated histone marks studied. Previous research by Lu et al. also demonstrated that histone methylation on the *Dlk1* promoter was enhanced at H3K4 and decreased at H3K27 in conjunction with an increase in *Dlk1* expression in Trim28 (a crucial regulator of development and differentiation)-knockdown cells [28]. By correlating the levels of Dlk1 histone *markers* with foetal growth parameters, we observed a negative correlation between H3K4me3 levels, head circumference, crown–rump length, and foetal weight. Regarding promoter region histone modifications in *Grb10,* high levels of the activating marker H3K4me3 in the BDFO transient group, along with all the sustained dietary groups, were also detected in F1 placental tissue. Correspondingly, we found a significantly increased level of *Grb10* expression in the F1 generation in these groups, viz. BDFO transient, BDFD, BDFN, and BDFO sustained groups. A study by de Sá Machado et al*.* also revealed differential enrichment of H3K4me3 and H3K27ac signatures for the imprinted DMRs of *Grb10* in tissues with monoallelic expression [29]. However, the increased levels of the repressive marks H3K27me3 and H3K9me3 found under BDFD-susceptible conditions in the F1 generation did not correlate with the expression of *Grb10*. Yamasaki-Ishizaki et al. reported that tissue-specific imprinting of mouse *Grb10* is influenced by H3K27 trimethylation [30]. Research by Monk et al. also verified that the paternal allele of *Grb10* had both H3K4 and H3K27 methylation [31]. According to our data, there was a significant increase in *Grb10* expression in the groups under sustained dietary conditions, particularly in the folate-oversupplemented and vitamin B12-deficient groups. Additionally, we detected a decrease in the levels of repressive histone marks in this group (H3K9me3 and H3K27me3). Our study examined the correlation between histone modifications and foetal growth parameters. The results showed a negative correlation between the levels of histone markers and all the parameters that were examined.

*Dlk1* is widely expressed during tissue regeneration and the development of organisms [32]. In the present study, vitamin B12 deficiency reduced the expression of the *Dlk1* gene regardless of the amount of folic acid available in the maternal placenta of the F0 generation. However, in F2 mice, the sustained state of both vitamin deficiencies (BDFD) led to increased expression in the placenta. *Dlk1* expression in the early growth period regulates fetal development; studies have shown that *Dlk1* mRNA levels at the time of birth in the placenta appear to be lower in the placentas of infants born at a younger gestational age than in those of standard-sized children [33]. In agreement with these findings, Kappil et al*.* proposed that there is a positive correlation between higher birth weight and placental expression of *Dlk1* [34]. Conversely, Schrey et al*.* reported that pregnancy complications are linked to decreased maternal levels of circulating *Dlk1* [35]. Additionally, prior research has shown that the foetus is the source of the high concentration of *Dlk1* that is circulated in mothers during late pregnancy; when the mother does not have fetally derived *Dlk1,* her fasting response is compromised [36]. In the present study, male and female pups from groups with vitamin B12 deficiency either alone or in conjunction with folate deficiency or from excessive supplementation in the F1 generation showed an increase in *Dlk1* expression in fetal tissues (namely, the liver and brain). Transient circumstances resulted in decreased expression of *Dlk1*, whereas prolonged folate shortage or oversupplementation along with vitamin B12 insufficiency increased *Dlk1* expression in the fetal liver tissue of the F2 generation. Although research has shown that *Dlk1* is not expressed in the average adult mammalian liver, it is still believed that *Dlk1* is a marker of bipotential hepatocytes, which can differentiate into mature hepatocytes and create de novo bile ducts [37–39]. This finding indicates the role of *Dlk1* expression in liver regeneration. In the fetal brain, the expression of *Dlk1* followed the same trend as that in the fetal liver, with increased Dlk1 expression in both the F1 generation and sustained F2 groups in both the BDFD and BDFO groups.

In contrast to that of *Dlk1, Grb10* expression can arise from both parental alleles. Paternal uniparental disomy causes fetal overgrowth, while maternal uniparental disomy causes growth failure. It is produced entirely from the maternal allele in mice, but it has a distinct expression pattern in humans that is paternally specific to the human fetal brain. Our study revealed a significant decrease in the transcription of the *Grb10* gene in the placental tissue of the F0 generation in the BDFD, BDFN, and BDFO groups. In the F1 generation, *Grb10* expression increased in all the sustained dietary groups and the BDFO transient groups. In a study by Mukhopadhyay et al*.*, placental *Grb10* expression was positively correlated with placental weight and neonatal parameters, particularly in males. This finding implies that foeto-placental development is regulated by placental *Grb10* [40]. Another study reported that mothers with peripheral tissue deficiency of *Grb10* had larger brood sizes at the same time as their placental and embryonic weights decreased, indicating that *Grb10* influences many pathways related to the distribution of maternal resources [41].

The expression of hepatic *Grb10* was elevated in fetal tissues among females in the BDFO and BDFD groups in the F1 generation, indicating that its levels were not impacted by vitamin B12 deficiency alone. Nevertheless, it was discovered that in the F2 generation, the expression only increased in the sustained group of both male and female liver tissue. The fetal brains of the male and female fetuses in the BDFO group showed a similar pattern of elevated expression. Notably, BDFO was more highly expressed in the brains of fetuses of both sexes in the F1 generation. Overall, under combined vitamin B12 deficiency, the expression of *Grb10* increased in maternal and fetal tissues (liver and brain) but not in maternal placenta in the F0 generation. Studies have also shown that disruption of *Grb10* expression due to transcriptional and epigenetic changes can lead to type II diabetes [25]. Previous research conducted by Shiura et al. demonstrated that postnatal growth retardation and insulin resistance are caused by the overexpression of Grb10, which negatively regulates the insulin signalling pathway [42].

Fetal growth abnormalities are attributed to changes in fetal gene expression, possibly due to epigenetic alterations. The expression of the investigated genes, *Grb10* and *Dlk1*, in the placental tissue of the F0 generation of our mouse model was downregulated under various dietary combinations of folic acid with B12 deficiency and was not related to changes in CpG methylation levels. However, nutritional influences can affect the bioavailability and transfer of methyl groups; when DNA methylation patterns are formed during pregnancy in the fetal genome, long-lasting alterations to the fetal genome may occur. We observed a reduction in *Grb10* expression in placental cells after treatment with different combinations of low vitamin B12 and folic acid (BDFD, BDFN, and BDFO). This downregulation is similar to the trend in the expression of these genes observed in placental tissue of the F1 generation in animal models. We further examined the expression of fetal growth-related genes in placental cells upon treatment with 5-azacitidine. We observed significant upregulation of *Dlk1 and Grb10* mRNA expression in 5-aza-treated cells compared to untreated cells in all the vitamin groups. The increase in expression was consistent for both genes, particularly under low vitamin B12 and high folic acid conditions. Therefore, CpG, or DNA methylation, may be crucial for controlling placental cell expression of imprinted genes linked to foetal growth.

## Conclusion

The data we have presented revealed the important role of H3K4 trimethylation in vivo in the upregulation of the expression of imprinted genes when there is an imbalance of reduced vitamin B12 and excess folic acid levels. However, in our study, we selected a defined region in the promoters of the genes to study CpG methylation, which might not be a regulatory region. One limitation of our study is that we did not determine the methylation levels in the whole promoter regions of the genes. In summary, our findings shed light on the intimate connection between an imbalanced diet (vitamin B12 and folic acid) and fetal growth in terms of the regulation of gene expression by chromatin. Apart from the experimental investigations of the suggested mechanisms, our research emphasizes the significance of a balanced vitamin diet recommendation for both male and female parents.

## Supplementary Information

Supplementary Material 1.

## Data Availability

The data supporting the findings of this study are found in the article and the supplementary material. The corresponding author will make all relevant raw data available upon reasonable request.
