# Supplementary material for: Enrichment of trimethyl histone 3 lysine 4 in the Dlk1 and Grb10 genes affects pregnancy outcomes due to dietary manipulation of excess folic acid and low vitamin B12
Source: Biol Res. 2024 Nov 14;57:85. doi: 10.1186/s40659-024-00557-3 (PMC11562088; doi:10.1186/s40659-024-00557-3)
Supplement: Supplementary file 1 — Supplementary Material 1. [file 40659_2024_557_MOESM1_ESM.docx]

Table 1: Composition of the diet

| **Ingredients** | **g/kg** |
| --- | --- |
| Caesin | 180 |
| Starch | 265 |
| Sucrose | 265 |
| Cellulose | 110 |
| Salt Mixture | 60 |
| Choline | 2 |
| Vitamin Mixture | One tablet |
| Folic acid | 2 |
| Corn oil | 110 |

*In normal folate diet, 1% succinyl-sulfathiazole was used with 2 mg/kg folic acid. In folate deficient diet no folic acid was added and 1% succinyl-sulfathiazole was used. In folate over-supplemented diet, diet with 8mg/kg folic acid and 1% succinyl-sulfathiazole was used. The content of vitamin b12 in deficient group was 0 mg/kg diet, in b12 normal group was 0.025mg/kg diet and in b12 over-supplementation group it was 0.1 mg/kg diet.*

Table 2: Primer sequences along with their annealing temperatures used for bisulfite sequencing are given below”

| **Gene** | **Primer sequence: 5’-3’** | **No. of CpGs analyzed** | **Annealing temperature (ºC)** |
| --- | --- | --- | --- |
| *Dlk1* | F: TGTATTTGTGATTATGTGTATTTTTTATAT  R: TCTCAAAAACCAAACCAAAC | 12 | 55 |
| *Grb10* | F: ATTATGATTTTAGTATGTTTTTTTT  R: AAACTCCAAAACCCTTTTTCTAAAC | 23 | 57 |

Table 3: Primers sequences and conditions used in the ChIP-qPCR

| **Gene** | **Primer sequences: 5’-3’** | **Annealing temperature (°C)** |
| --- | --- | --- |
| *Dlk1* | F: CCCAGGTGAGCTTCGAGTG  R: GGCGGTAGGTGAGCCCATA | 68.4 |
| *Grb10* | F: GTTACATGCGCCAACACTGG  R: CCGCGATCATTCGTCTCTGA | 68.4 |

| **Gene** | **Primer sequences: 5’-3’** | **Amplicon**  **Size** | **Annealing temperature (ºC)** |
| --- | --- | --- | --- |
| *Dlk1* | F-CACGGACTCTGTGGAGAACC  R-AGCAGGCCCGAACATCTCTA | 85bp | 60.7 ºC |
| *Grb10* | F-CTCTCCAGCTTTGGCGCT  R-GCTTCACTGAGAGGACCCAG | 120bp | 60 ºC |

Table 4: Primer sequences and conditions for qPCR

| **Gene** | **Primer sequences: 5’-3’** | **Annealing**  **temperature (°C)** | **Amplicon**  **length bp** |
| --- | --- | --- | --- |
| *Dlk1* | F: CAACCAACAGCTGTACCCCTAAC  R: GCAGCGGCAACGGAAGT | 78 | 80bp |
| *Grb10* | F: ACAGGATCATCAAGCAACAAGGT  R: TACGAACGCCTTTGGATTACTCT | 80 | 79bp |

Table 5: Primer sequences and conditions of qPCR for *in-vivo* studies:
